# Deep Learning-Based Risk Assessment and Prediction of Cardiac Outcomes Using Single-Lead 24-Hour Holter-ECG in Patients with Heart Failure or Myocardial Infarction

**DOI:** 10.3390/jcm14207209

**Published:** 2025-10-13

**Authors:** Ju Youn Kim, Kyung Geun Kim, Sunghoon Joo, Mineok Chang, Juwon Kim, Kyoung-Min Park, Young Keun On, June Soo Kim, Young Soo Lee, Seung-Jung Park

**Affiliations:** 1 Division of Cardiology, Department of Internal Medicine, Heart Vascular and Stroke Institute, Samsung Medical Center, Sungkyunkwan University School of Medicine, Seoul 06351, Republic of Korea; 2 VUNO Inc., Seoul 06541, Republic of Korea; 3 Department of Bioengineering, Stanford University, Stanford, CA 94305, USA; 4 Daegu Catholic University Medical Center, Daegu 42472, Republic of Korea

**Keywords:** heart failure, myocardial infarction, cardiac death, ventricular arrhythmia, deep learning, ejection fraction, heart rate turbulence, T-wave alternans

## Abstract

**Background:** Deep learning (DL) models using Holter-ECG may enhance risk stratification after heart failure (HF) or myocardial infarction (MI). **Objective:** To evaluate the prognostic performance of a Holter-based DL model for predicting major adverse cardiac events (MACE), compared with conventional noninvasive markers. **Methods:** In the K-REDEFINE study, 1108 patients with acute MI or HF underwent 24 h Holter monitoring. A DL model was trained using raw Holter-ECG data and tested for predicting a composite of cardiac death and ventricular arrhythmias. Its performance was compared with heart rate turbulence (HRT), T-wave alternans (TWA), and ejection fraction (EF). **Results:** During follow-up, 56 adjudicated cardiac deaths (1.18%/yr) and 21 ventricular arrhythmias (0.44%/yr) occurred. The DL model showed an area under the receiver operating characteristic curve (AUROC) of 0.74 (95% CI, 0.70–0.77) for the composite outcome, improving to 0.77 (0.74–0.81) when combined with EF. In comparison, HRT and TWA showed lower AUROCs of 0.62 and 0.55, respectively. For cardiac death alone, the AUROC reached 0.79, further improving to 0.82 with EF. Model-derived risk stratification revealed a seven-fold increase in cardiac death risk in the high-risk group compared to the low-risk group (HR 7.47, 95% CI 2.24–24.96, *p* < 0.001). This stratification remained particularly effective in patients with EF > 40%. **Conclusions:** A DL algorithm trained on single-lead Holter-ECG data effectively predicted cardiac death and ventricular arrhythmia. Its performance surpassed conventional markers and was further enhanced when integrated with EF, supporting its potential for noninvasive, scalable risk stratification.

## 1. Introduction

Heart failure (HF) and myocardial infarction (MI) are leading causes of major adverse cardiac events (MACE), including ventricular arrhythmias and cardiac death [1,2]. Although severe left ventricular (LV) dysfunction remains the primary indication for prophylactic implantable cardioverter-defibrillator (ICD) therapy, many sudden cardiac deaths (SCD) still occur in patients with preserved or mildly reduced LV systolic function [3]. This highlights the need for improved, noninvasive risk stratification tools beyond conventional ejection fraction (EF)–based criteria.

Recent advances in machine learning (ML) have shown promise in various aspects of HF management, including diagnosis, phenomapping, and prognostication. Artificial intelligence (AI)-driven risk prediction models based on heart rate variability and electrocardiogram (ECG) data have been proposed for prognostic use in HF populations [4]. In acute HF (AHF) patients, the deep learning (DL)-based AI algorithm integrates echocardiographic, laboratory, and ECG data to estimate both in-hospital and long-term mortality more accurately than the existing risk scores [5]. Similarly, the MARKER-HF risk score, developed from various clinical and laboratory variables, has demonstrated utility in stratifying mortality risk among HF patients [6].

However, the clinical applicability and performance of many existing models are limited by their dependence on a large number of input features, retrospective data sources, and associated challenges such as lots of missing values and heterogeneity (e.g., lots of outliers).

The K-REDEFINE (Korean noninvasive Risk Evaluation study for sudden cardiac DEath From INfarction or heart failurE) is a prospective, nationwide multicenter registry study designed to assess the predictive value of Holter-based noninvasive variables, such as heart rate turbulence (HRT) and T-wave alternans (TWA) in patients with HF or MI. Building on our previous findings that abnormal HRT predicts adverse outcomes, including cardiac outcomes [7], the present study aimed to investigate whether a DL algorithm trained on 24 h Holter-ECG data could predict MACE, and to compare its performance against conventional noninvasive markers in a well-characterized prospective cohort.

## 2. Methods

### 2.1. Study Population

This study used data from the K-REDEFINE study cohort, which is a prospective, multi-center, observational registry conducted at 25 tertiary cardiovascular centers across South Korea. The study’s design and specific details, including acquisition and analysis of ambulatory ECG data, have been previously described [7,8]. Eligible participants were adult patients (≥19 years) hospitalized for acute MI or acute HF between September 2015 to December 2019. Individuals with persistent or permanent atrial fibrillation, those dependent on ventricular pacing, patients with an estimated survival of less than one year, or those diagnosed with end-stage renal disease were not considered eligible for inclusion in this study. Additionally, patients were excluded if their Holter-ECG raw data could not be successfully extracted in MIT format for DL-based analysis. All patients underwent 24 h ambulatory ECG monitoring within 3 months of hospitalization and were in sinus rhythm at the time of enrollment. Holter recordings were obtained using a 2 or 3-lead SEER Light Digital Holter monitor (GE Healthcare Inc., Milwaukee, WI, USA) with a sampling rate of 125 Hz. Raw wave signals were exported in MIT format using the MARS 8000 Holter analysis system (GE Healthcare Inc., Milwaukee, WI, USA) for further analysis. This study protocol was approved by the Institutional Review Board (IRB) of Samsung Medical Center in the Republic of Korea (IRB number: 2015-08-086), and written informed consent was obtained from all participants.

### 2.2. Data Collection

Data collection was prospectively performed by attending physicians using a web-based electronic case report form at each participating center. Raw Holter data were transferred to the core laboratory (Samsung Medical Center), where they underwent standardized analysis protocols as previously described [7,8]. For the present deep learning analysis, an additional data conversion pipeline into MIT format was implemented to ensure uniform data structure across centers and to minimize noise-related variability.

### 2.3. Overview of the AI Model

The intrinsic characteristics of Holter recordings pose unique challenges for deep learning applications. In particular, the extensive duration of ECG recordings from Holter monitors often makes them impractical for deep learning processing, as most existing neural network architectures are not designed to handle such lengthy data. Additionally, the limited availability of data samples, due to the challenges of Holter monitoring, further complicates the training of deep neural networks, which typically require large datasets to learn effectively. To address these limitations, we utilized a pretrained Transformer model trained on an internal dataset at VUNO Inc [9,10]. With the pretrained Transformer, extremely lengthy Holter-ECG signals were encoded into latent vectors with greatly reduced dimension, suitable for downstream analysis. Specifically, the pretraining was performed in two steps. First, we pretrained the Masked Autoencoder (MAE) using standard 12-lead, 10 s ECGs, with random masks applied to ECG waveforms [11]. Subsequently, the MAE was fine-tuned using supervised learning with left ventricular ejection fraction (LVEF) as a surrogate label, based on its utility as an indicator of global cardiac function. From the dataset used for the MAE pretraining (a standard 12-lead, 10 s ECGs), the middle 9 s window ECG signals were taken for pretraining. In addition, to enhance generalizability, we trained a single-lead model by randomly selecting the input lead during training, considering that the lead positions of the Holter monitor slightly differ from the standard 12-lead ECG. With this encoder, we divided 24 h Holter-ECG into 9600 non-overlapping 9 s segments, with each segment encoded into a single latent value, obtaining size 9600 latent vectors per patient. For the cases where the Holter-ECG recording is longer or shorter than 24 h, we encode them as is, obtaining a latent vector that slightly differs in size. During training, the latent vectors are zero-padded to the longest vector for efficient batching. We utilized only the V1 (mV1) lead because the V1 lead was always used for Holter recording in all participating centers.

After we encoded all the Holter-ECGs, we trained a downstream classifier with our aimed outcomes as labels. The downstream classifier was a compact 1-D ResNet operating on the feature sequence output by the pretrained Transformer [12]. The network employed an initial 7 × 1 convolutional layer, followed by BatchNorm, ReLU, a 3 × 1 max-pooling layer, and four residual stages, each consisting of one BasicBlock with channel widths of [64, 128, 256, 512]. Each BasicBlock comprised two 3 × 1 convolutions with BatchNorm followed by ReLU. After the residual blocks, global average pooling and dropout (*p* = 0.2) are applied before feeding the output into a fully connected layer for prediction. The ResNet was trained using the Adam optimizer with a learning rate of 0.002 and a batch size of 8, with a separate validation set used to determine convergence. Additionally, Holter signals were converted from MIT format and band-pass filtered (0.67–40 Hz) before Transformer encoding.

Model performance was evaluated using five-fold cross-validation due to the limited dataset size. To mitigate overfitting, given the modest number of labeled outcome events, outcome-supervised training was restricted to a lightweight classifier operating on low-dimensional latent vectors, with all parameters of the pretrained encoder fixed, and patient-wise five-fold cross-validation employed to ensure stability.

LVEF values were also used during prediction when available. When combining LVEF values with the probability scores predicted by the neural network, we categorized patients into three LVEF bins: HFrEF (≤40%, a normalized risk score = 1), HFmrEF (41–49%, score = 0.1), and HFpEF (≥50% or missing, score = 0). The final risk score is then computed as the average of the probability score from the deep neural network and the normalized risk score value computed from the LVEF values.

### 2.4. Study Outcomes

The primary outcome of the study was the composite of cardiac death and ventricular arrhythmias occurring during a 3-year follow-up period. All deaths were considered to be noncardiac unless a definitive cardiac cause could be identified. Ventricular tachyarrhythmia included documented cases of ventricular fibrillation and ventricular tachycardia that resulted in SCD or aborted SCD. In patients with an ICD, it also encompassed appropriate ICD shocks or antitachycardia therapy. SCD was defined as unexpected death due to cardiac causes that occurs within a short time period (within 1 h of symptom onset or unwitnessed death during sleep) [7,8]. The predictive performance of the DL model was evaluated with respect to this composite outcome and compared against conventional noninvasive parameters, including HRT, TWA, and LVEF. We also assessed the performance of the DL model in combination with LVEF.

Pre-specified subgroup analyses were conducted according to baseline LV systolic function, categorized as preserved or mid-range EF (>40%) versus reduced EF (≤40%), as well as by underlying condition (HF versus MI).

### 2.5. Statistical Analysis

Continuous variables are presented as mean values with corresponding 95% confidence intervals (CIs), while categorical variables are expressed as counts and percentages. Group comparisons were performed using the Mann–Whitney U test for continuous variables and Fisher’s exact test for categorical variables, as appropriate.

The discriminative performance of the DL model and comparator variables was evaluated by calculating the area under the curve (AUC) using the receiver operating characteristic (ROC) curve. All statistical analyses were conducted using SPSS software, version 27.0 (SPSS Inc., Chicago, IL, USA).

## 3. Results

### 3.1. Clinical Characteristics

A total of 1108 patients were included in the analysis. This number was slightly lower than in our previous report of the K-REDEFINE cohort (n = 1116), because Holter-ECG raw data from 8 patients could not be successfully extracted into the MIT format required for the deep learning analysis [7]. The mean age was 60.8 ± 12.9 years, and 844 patients (76.2%) were male. Among the cohort, 223 patients (20.1%) were enrolled for acute HF, and 885 (79.9%) for acute MI. The mean interval between the index event (HF or MI) and 24 h ambulatory ECG recording was 6.8 ± 16.5 days. The average duration of Holter monitoring was 1860.9 min, yielding a total of 13,746,135 ECG segments for the analysis.

The mean LVEF was 48.2 ± 16.7%. Based on the pre-specified categories, 529 patients (47.7%) had preserved EF (≥50%), 291 (26.3%) had mid-range EF (41–49%), and 258 (23.3%) had reduced EF (≤40%). Additional baseline characteristics are summarized in Table 1 and have been reported previously [7].

### 3.2. Clinical Events and Predictive Performance

During follow-up, 56 cardiac deaths (1.18%/yr) and 21 ventricular arrhythmias (0.44%/yr) occurred, with a total of 70 patients (1.48%/yr) experiencing the composite endpoint. Of the 56 cardiac deaths observed, 19 were classified as SCD. In addition, 12 patients experienced aborted SCD events—9 due to ventricular fibrillation and 3 due to ventricular tachycardia—that were successfully resuscitated by cardiopulmonary resuscitation. A total of 25 patients underwent defibrillator implantation, including 17 with an ICD and 8 with a cardiac resynchronization therapy-defibrillator. Among these, 4 patients received a total of 9 appropriate ICD shock therapies. Notably, the number of patients with ventricular tachyarrhythmias in the present analysis (n = 21) was slightly lower compared with our previous report (n = 23), owing to the exclusion of two patients whose Holter-ECG data could not be processed in MIT format [7].

**Figure 1:**
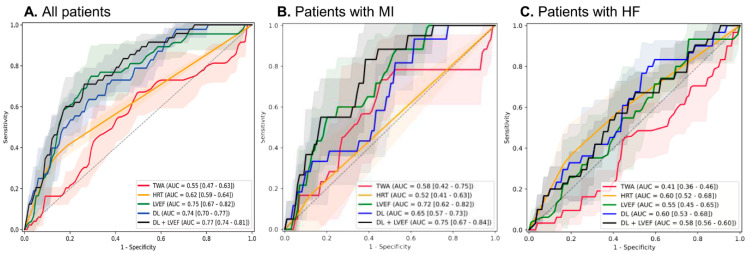
Receiver operating characteristic curves for the performance of the AI model and other variables for the composite of cardiac death and ventricular arrhythmia. (**A**) Total population (**B**) MI population (**C**) HF population.

For predicting the composite outcome at 3 years, the DL model achieved an AUROC of 0.74 (95% CI, 0.70–0.77), with high specificity (0.96) but modest sensitivity (0.27). The corresponding positive likelihood ratio was 6.8, and the negative likelihood ratio was 0.76. When combined with LVEF, performance improved further (AUROC 0.77, 95% CI, 0.74–0.81). In contrast, traditional Holter-based markers showed lower discriminative ability (HRT with 0.62 [0.59–0.64] and TWA with 0.55 [0.47–0.63]), while LVEF alone performed at 0.75 (0.67–0.82) (Figure 1A, Table 2).

Subgroup analyses revealed consistent trends. In patients with MI, the DL model alone reached an AUROC of 0.65 (0.57–0.73), which improved to 0.75 (0.67–0.84) with EF. HRT and TWA were inferior (0.52 [0.41–0.63] and 0.58 [0.42–0.75], respectively), while LVEF alone performed at 0.72 (0.62–0.82) (Figure 1B). In the HF subgroup, DL yielded an AUROC of 0.60 (0.53–0.68), similar to HRT (0.60 [0.52–0.68]), and better than TWA (0.41 [0.36–0.46]) or LVEF (0.55 [0.45–0.65]); however, the combination of DL and LVEF showed minimal additive value (0.58 [0.56–0.60]) (Figure 1C).

**Figure 2:**
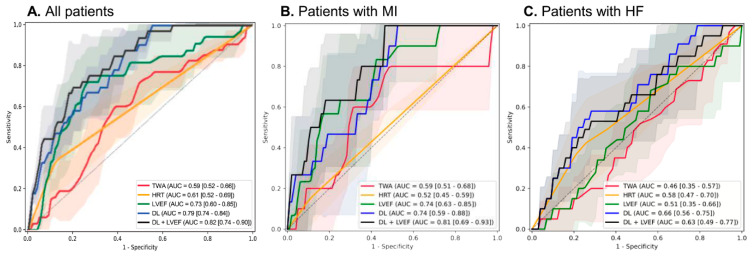
Receiver operating characteristic curves for the performance of the AI model and other variables for cardiac death. (**A**) Total population (**B**) MI population (**C**) HF population.

When focusing on cardiac death alone, the DL model demonstrated stronger predictive power (AUROC 0.79 [0.74–0.84], specificity 0.95, sensitivity 0.38), further increasing to 0.82 (0.74–0.90) with LVEF (Figure 2A, Table 3). Again, DL outperformed HRT (0.61 [0.52–0.69]), TWA (0.59 [0.52–0.66]), and LVEF (0.73 [0.60–0.85]). This pattern was similar across MI and HF subpopulations, with DL combined with LVEF consistently showing the best performance (Figure 2B,C).

**Figure 3:**
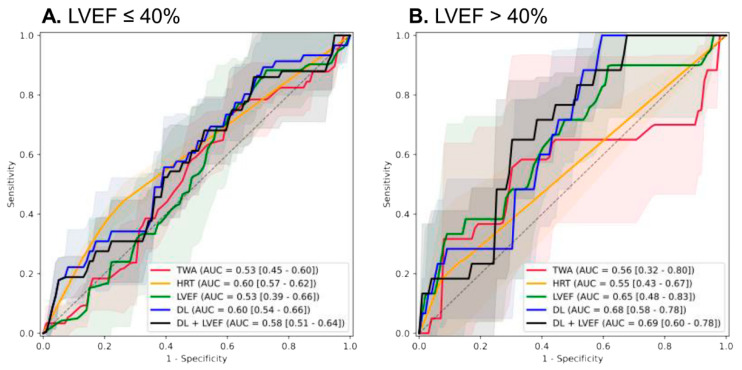
Receiver operating characteristic curves for the performance of the AI model and other variables for composite of cardiac death and ventricular arrhythmia. (**A**) severely reduced left ventricular ejection fraction (**B**) preserved or mid-range reduced left ventricular ejection fraction.

Importantly, subgroup analysis by baseline LVEF revealed superior performance of the DL model in patients with preserved or mid-range EF [AUROC 0.68 (0.58–0.78)] compared to those with severely reduced EF [AUROC 0.60 (0.54–0.66)], highlighting its particular value in less advanced systolic dysfunction (Figure 3).

In an exploratory analysis, the presence of NSVT during 24 h Holter monitoring was also evaluated as a conventional marker alongside HRT and TWA. NSVT was observed in 56 patients (4.8%) and demonstrated low predictive performance for composite outcomes (sensitivity 0.06, specificity 0.95) and was not significantly associated with cardiac death (adjusted HR 1.47, 95% CI 0.53–4.08) (Supplementary Tables S1 and S2). By contrast, abnormal HRT was strongly associated with cardiac death (adjusted HR 3.27, 95% CI 1.81–5.90), whereas abnormal TWA showed a weaker, non-significant trend (adjusted HR 1.60, We changed the figure. 95% CI 0.94–2.73). Notably, patients classified as high risk by the DL-based score had a markedly increased risk (adjusted HR 7.31, 95% CI 2.19–24.47).

### 3.3. Hazard Across Model Probability Risks

**Figure 4:**
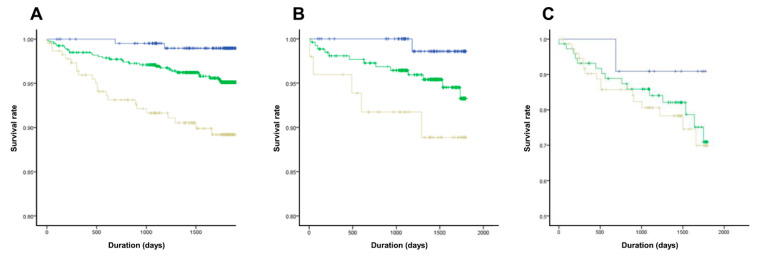
Kaplan–Meier curve according to the risk group for cardiac death (**A**) Total population (**B**) preserved or mid-range reduced left ventricular ejection fraction (**C**) severely reduced left ventricular ejection fraction (Blue indicates low-risk, green indicates intermediate-risk, and yellow indicates high-risk group).

In our cohort, patients were stratified into three groups according to the model-derived probability of cardiac death: low-risk (<0.3), intermediate-risk (0.3–0.6), and high-risk (≥0.6). As shown in Figure 4, Kaplan–Meier analysis revealed significant differences in survival among the groups (log-rank *p* < 0.001). Patients in the high-risk group had a substantially increased risk of cardiac death compared to those in the low-risk group (hazard ratio [HR] 7.47, 95% confidence interval [CI] 2.24–24.96).

Subgroup analysis by baseline LVEF showed a significant difference in survival between risk groups in patients with LVEF > 40% (log-rank *p* = 0.039), but not in those with LVEF ≤ 40% (log-rank *p* = 0.552).

## 4. Discussion

In this study, we investigated the prognostic performance of a DL-based AI algorithm trained on 24 h Holter-ECG data to predict cardiac death and ventricular arrhythmia leading to aborted SCD. The DL model outperformed conventional Holter-based markers such as TWA and HRT, and its predictive performance was further enhanced when combined with LVEF. These findings are particularly notable given the prospective design of the cohort and the rigorous adjudication of clinical outcomes.

The model’s predictive power was most evident for cardiac death, with an AUROC of 0.79 (increasing to 0.82 with LVEF), suggesting its potential clinical utility. Risk stratification based on model-derived probabilities revealed a seven-fold increase in cardiac death risk in the high-risk group compared to the low-risk group. Importantly, this stratification remained consistent across etiologies of HF, showing improved discrimination particularly in patients with ischemic HF, and in those with preserved or mid-range EF, where traditional EF-based assessment alone is known to perform suboptimally.

### 4.1. Challenges in Risk Stratification for Sudden Cardiac Death

SCD is one of the leading causes of death in both ischemic and non-ischemic cardiomyopathy. However, effective risk stratification remains limited [13]. Current guidelines recommend ICD therapy primarily in patients with ischemic HF and reduced EF [14], based on evidence of mortality reduction [15]. Nevertheless, a recent study using pooled cohort analysis involving over 140,000 post-MI patients showed that LVEF alone had poor predictive power for SCD. Moreover, incorporating additional clinical variables such as demographics, medical history, biomarkers, ECG, echocardiography, or cardiac magnetic resonance imaging did not meaningfully improve the predictive performance [16]. In this context, the PRESERVE-EF study proposed a two-step strategy in which noninvasive risk factors are used to guide programmed ventricular stimulation (PES) for arrhythmic risk stratification in post-MI patients with preserved EF, representing an important benchmark in primary prevention SCD research [17]. However, this approach remains invasive, requires specialized facilities and expertise, and is not easily repeatable for longitudinal monitoring. Patient acceptance may also be limited, and its predictive performance in certain subgroups—such as those with non-ischemic cardiomyopathy—remains uncertain. In contrast, our DL-based algorithm offers potential complementary value, although our study did not include PES data and thus could not directly compare our approach with this strategy. Specifically, it enables fully noninvasive, repeatable risk assessment by analyzing continuous 24 h Holter-ECG data, allowing large-scale screening without procedural risk. This noninvasive nature may facilitate broader clinical application, particularly in settings where PES is not readily available or feasible. Further prospective studies directly comparing DL-based risk stratification with PES-guided approaches are warranted to clarify their relative and combined value in clinical practice.

In non-ischemic cardiomyopathy, the role of ICD for primary prevention is even more controversial [18,19]. Risk stratification in this population is complicated by heterogeneity of pathophysiologic mechanisms and clinical phenotype, such as gene mutation (e.g., LMNA) or infiltrative disorders (e.g., sarcoidosis) [20], which challenge the development of universal management strategies, as demonstrated by limitations of relying upon EF for risk assessment [19,21]. This challenge is further compounded by the fact that the absolute number of SCD cases is higher in patients with mildly reduced or preserved EF than in those with severely reduced EF.

### 4.2. Personalized Risk Prediction Using Deep Learning

Given these limitations of conventional risk stratification, there is an unmet need for more individualized approaches to risk stratification, particularly in patients with non-ischemic HF and those with preserved or mid-range EF. In response to this growing demand, AI research has been increasing in this patient group. Building on this momentum, recent advances in ML and AI have enabled the extraction of predictive patterns from complex cardiovascular datasets, often surpassing conventional statistical models in accuracy. For example, an ML-based approach utilizing P, QRS, and T wave features from high-risk treadmill exercise tests has been shown to predict obstructive coronary artery disease with high precision [22]. Likewise, a deep learning model using multimodal data was successfully applied to predict short-term mortality in patients with acute pulmonary embolism [23]. These studies illustrate the versatility of AI-based tools across different cardiovascular conditions, supporting their potential to enhance current risk stratification strategies. While prior DL studies have illustrated the feasibility of ECG-based risk prediction, producing tools with robust individual-level performance remains scarce [16]. Our study, leveraging a prospective national cohort with adjudicated cardiac deaths, demonstrated that a DL-based Holter-ECG model may offer a noninvasive and repeatable method that could complement existing clinical workflows and facilitate large-scale screening for arrhythmic risk. At the same time, the model showed only modest sensitivity (27%), underscoring its limitations as a stand-alone screening tool. However, its positive likelihood ratio was high (6.8), indicating that patients classified as high risk truly carried a markedly increased probability of adverse events. This suggests that the model may be most valuable not as a general screening tool but as a complementary means of pinpointing a subset of particularly high-risk individuals who warrant intensified surveillance and proactive management.

A further strength of our approach lies in its technical implementation. By employing pretrained encoders to capture latent information embedded within continuous ECG signals, the model was able to identify hidden features relevant to risk prediction while overcoming the analytic challenges posed by lengthy 24 h Holter recordings. This methodological advance enabled efficient large-scale application without compromising predictive performance.

Importantly, our cohort included patients with both ischemic and non-ischemic HF. Among these patients, those classified as high-risk by the DL model had a seven-fold increase in cardiac death risk compared to the low-risk group. Subgroup analyses further revealed that the incremental predictive value of the model was most pronounced in patients with preserved or mildly reduced EF (>40%). This finding likely reflects that in patients with EF ≤ 40%, the high baseline risk already provides strong prognostic information, leaving limited room for improvement, whereas in patients with EF > 40%, conventional EF thresholds fail to adequately identify vulnerable individuals. In this context, a DL-based Holter approach could complement current ICD implantation criteria, which are largely EF-driven, by identifying high-risk individuals with EF > 40% who are not captured by conventional thresholds. For such patients, intensified surveillance and proactive management may be particularly warranted.

Another strength of our approach lies in its focus on cardiac-specific outcomes, such as cardiac death (including SCD) and ventricular arrhythmia. This outcome specificity may enhance clinical relevance and aid in developing personalized management strategies.

Finally, the DL model operates on continuous single-lead ECG data without requiring a standard 12-lead ECG or additional clinical variables. This feature is especially meaningful in the current landscape of expanding use of wearable and patch-based ECG monitors, which predominantly record single-lead signals. When integrated into existing clinical workflows, such approaches could serve as a complementary tool alongside conventional risk factors, imaging modalities, biomarkers, and electrophysiological testing, thereby contributing to a more comprehensive and individualized strategy for SCD prevention.

### 4.3. Limitation

This study has several limitations. First, due to the low incidence of ventricular arrhythmia events, we primarily analyzed a composite outcome comprising ventricular arrhythmia and cardiac death. This approach ensured adequate statistical power but may have obscured potential differences in predictive performance between arrhythmic and mortality outcomes. Separate analyses confirmed robust performance for cardiac death alone, whereas ventricular arrhythmia–specific prediction was underpowered. Moreover, the modest total number of outcome events (70 among 1108 patients) inevitably widened confidence intervals and increased the risk of model instability, despite safeguards such as dimensionality reduction, pretraining, and patient-wise cross-validation. Second, the model was developed and evaluated entirely within a single national cohort, without external validation in independent datasets. This limitation restricts the ability to generalize the findings to other populations, healthcare systems, and device configurations, and raises the possibility that the observed performance may, in part, reflect cohort-specific characteristics rather than true universal applicability. To address this, multicenter validation studies are currently under discussion within the K-REDEFINE consortium. These efforts are expected to further establish the robustness and broader clinical applicability of the proposed model. Third, the current DL model functions as a “black box,” offering limited interpretability into the specific ECG features driving its predictions. This limitation should be recognized as a major barrier to clinical adoption, since the lack of interpretability may reduce clinician confidence in applying the model to high-stakes decisions such as SCD risk stratification. Future iterations will incorporate explainability techniques, such as attention map-based explainability methods, saliency mapping, and Shapley Additive Explanations (SHAP), to highlight temporal and morphological ECG patterns most influential to the model’s predictions, thereby improving transparency, fostering clinician trust, and potentially uncovering novel mechanistic insights [24,25,26]. Fourth, our results were based on outcomes observed during a three-year follow-up period after enrollment. Since the study specifically aimed to evaluate whether Holter recordings obtained at the time of hospitalization for MI or HF—representing the acute phase—could predict long-term prognosis, we believe that the presented findings provide meaningful insights into early risk stratification. Nevertheless, cardiac risk is inherently dynamic and evolves with disease progression, therapeutic interventions, and changes in clinical status. Because we did not perform serial analyses using follow-up Holter recordings, we are unable to determine whether model performance remains stable over time or establish evidence regarding the appropriate frequency of repeated assessments. Future longitudinal studies with periodic Holter monitoring will be required to address this important question. Fifth, the incorporation of LVEF into the final risk score was based on coarse categorical stratification rather than continuous values, which may have limited the precision of risk integration. Therefore, more sophisticated strategies such as continuous EF modeling, data-driven cutpoints, and ensemble/meta-learning integration will be required in future work. Finally, the present study did not address practical considerations for real-world implementation of the algorithm, such as computational infrastructure, workflow integration with existing Holter analysis systems, cost-effectiveness, or regulatory pathways. While these issues were beyond the scope of our current investigation, they represent essential steps for clinical translation and should be systematically evaluated in future research.

## 5. Conclusions

In this prospective multicenter cohort, our DL–based AI algorithm, trained on continuous single-lead Holter-ECG data, effectively predicted cardiac death and ventricular arrhythmias leading to aborted SCD. The model outperformed conventional Holter-based markers, such as TWA and HRT. Notably, predictive performance was superior in patients with preserved or mid-range EF (≥40%) compared to those with reduced EF. These findings highlight the potential of AI-driven ECG analysis as a novel, noninvasive approach to identify high-risk subgroups among patients with HFmrEF or HFpEF—populations for whom current risk stratification tools remain inadequate.

## Figures and Tables

**Table 1:** Clinical characteristics of the study population.

	Total (n = 1108)
Age [years], mean(SD)	60.8 ± 12.9
Gender, male (%)	844 (76.2)
Body weight [kg], mean (SD)	67.2 ± 13.5
BMI, mean (SD)	24.8 ± 13.8
Medical history, n (%)	
Hypertension	606 (54.7)
Stroke	69 (6.2)
Diabetes	327 (29.5)
Vascular disease	128 (11.6)
CKD	74 (6.7)
EF [%], mean (SD)	48.2 ± 16.7
HFpEF	529 (47.7%)
HFmrEF	291 (26.3%)
HFrEF	258 (23.3%)

SD: standard deviation, BMI: body mass index, CKD: chronic kidney disease, EF: ejection fraction, HFpEF: heart failure preserved ejection fraction, HFmrEF: heart failure mildly reduced ejection fraction, HFrEF: heart failure reduced ejection fraction.

**Table 2:** Performance evaluation of our artificial intelligence model for composite of cardiac death and ventricular arrhythmias.

	Sensitivity	Specificity	PPV	NPV	F1 Score
DL model	0.27 (0.09–0.46)	0.96 (0.93–0.99)	0.30 (0.20–0.40)	0.96 (0.95–0.97)	0.23 (0.16–0.31)
DL with EF	0.36 (0.27–0.46)	0.92 (0.91–0.94)	0.19 (0.15–0.24)	0.97 (0.96–0.97)	0.25 (0.19–0.31)
EF	0.26 (0.11–0.42)	0.94 (0.91–0.97)	0.16 (0.06–0.26)	0.96 (0.96–0.97)	0.20 (0.08–0.31)

DL, deep learning; EF, ejection fraction; PPV, positive predictive value; NPV, negative predictive value.

**Table 3:** Performance evaluation of our artificial intelligence model for cardiac death.

	Sensitivity	Specificity	PPV	NPV	F1 Score
DL model	0.38 (0.25–0.51)	0.95 (0.93–0.97)	0.24 (0.19–0.29)	0.98 (0.97–0.98)	0.27 (0.21–0.32)
DL with EF	0.44 (0.28–0.61)	0.96 (0.94–0.98)	0.31 (0.22–0.40)	0.98 (0.97–0.99)	0.32 (0.26–0.38)
EF	0.30 (0.17–0.44)	0.93 (0.91–0.94)	0.12 (0.08–0.15)	0.97 (0.97–0.98)	0.17 (0.11–0.22)

DL, deep learning; EF, ejection fraction; PPV, positive predictive value; NPV, negative predictive value.

## Data Availability

The data on which this article is based are available in the article itself and in the Supplementary Material. Due to intellectual property constraints, sharing the code and model weights is restricted. However, restricted access to statistical analysis for verified researchers may be provided upon request for research purposes.

## Supplementary Materials

The following supporting information can be downloaded at: https://www.mdpi.com/article/10.3390/jcm14207209/s1, Table S1: Performance evaluation of NSVT for clinical outcomes. Table S2: Hazard ratios of cardiac death by variables.
