# Supplementary material for: Deep Learning-Based Risk Assessment and Prediction of Cardiac Outcomes Using Single-Lead 24-Hour Holter-ECG in Patients with Heart Failure or Myocardial Infarction
Source: J Clin Med. 2025 Oct 13;14(20):7209. doi: 10.3390/jcm14207209 (PMC12565440; doi:10.3390/jcm14207209)
Supplement: Supplementary file 1 [file jcm-14-07209-s001.zip › jcm-3915925-supplementary.pdf]

Supplementary Table S1. Performance evaluation of NSVT for clinical outcomes

**NSVT**					
Clinical outcomes	**Sensitivity**	**Specificity**	**PPV**	**NPV**	**F1 score**
Composite outcomes*	0.06	0.95	0.07	0.94	0.06
Cardiac death	0.07	0.95	0.07	0.95	0.07
Ventricular arrhythmia	0.05	0.95	0.02	0.98	0.03

*, included cardiac death and ventricular arrhythmia

NSVT, non-sustained VT; PPV, positive predictive value; NPV, negative predictive value

Supplementary Table S2. Hazard ratios of cardiac death by variables

	**Cardiac death**	
	HR (95% CI)	aHR* (95% CI)
Abnormal HRT	**4.26 (2.38-7.64)**	**3.27 (1.81-5.90)**
Abnormal TWA	**1.78 (1.05-3.02)**	1.60 (0.94-2.73)
LVEF<50%	**4.52 (2.27-9.00)**	**4.10 (2.06-8.17)**
NSVT	1.48 (0.53-4.09)	1.47 (0.53-4.08)
High risk (vs. Low risk) group based on DL-based risk score	**7.47 (2.24-24.96)**	**7.31 (2.19-24.47)**

*, adjusted hazard ratio for age, and sex.

HRT: heart rate turbulence, TWA: T-wave alternans, LVEF: left ventricular ejection fraction, NSVT: non-sustained ventricular tachycardia, DL: deep learning
